# Beyond Nutrients: NOVA-Defined Dietary Patterns in Crohn’s Disease and Healthy Adults

**DOI:** 10.3390/nu18071068

**Published:** 2026-03-27

**Authors:** Ayva Lewis, Thea Ulsamer, Laura Franco, Stephanie Gold, Natasha Haskey, Maitreyi Raman

**Affiliations:** 1 Division of Gastroenterology, Cumming School of Medicine, University of Calgary, Calgary, AB T2N 4Z6, Canada; 2 Department of Botany and Zoology, Faculty of Science, University of British Columbia, Vancouver, BC V6T 1Z4, Canada; 3 Division of Gastroenterology, Icahn School of Medicine at Mount Sinai, New York, NY 10029, USA; 4 Department of Biology, Irving K. Barber Faculty of Science, University of British Columbia-Okanagan, Kelowna, BC V1V 1V7, Canada

**Keywords:** inflammatory bowel disease, Crohn’s disease, NOVA, ultra-processed foods, diet quality

## Abstract

**Background:** Diet quality and food processing patterns are increasingly recognized as important determinants of Crohn’s disease (CD) risk and disease outcomes; however, direct comparisons with healthy populations using integrated nutrient- and processing-based frameworks remain limited. Therefore, we aim to quantify ultra-processed food (UPF) consumption using the NOVA classification, compare UPF intake between CD patients and healthy controls, and assess its association with diet quality indices. **Methods:** Baseline dietary intake data were analyzed from two randomized controlled trial cohorts: adults with mild to moderately active CD enrolled in the Crohn’s Disease Therapeutic Dietary Intervention (CD-TDI) trial (*n* = 64; NCT04596566), and healthy adults participating in the MAPMed study (*n* = 33, NCT06765369). Dietary intake was assessed using two non-consecutive 24 h recalls collected with the Automated Self-Administered 24-Hour Dietary Assessment Tool (ASA24^®^). Energy-normalized macronutrient and micronutrient intakes were compared with Dietary Reference Intakes (DRIs). Overall diet quality was evaluated using the Healthy Eating Index-2020 (HEI-2015), Alternate Mediterranean Diet score (aMED), and Dietary Inflammatory Index (DII). Foods were classified according to the NOVA food processing system to estimate total and proportional energy intake from UPFs (NOVA group 4). **Results:** Both the CD cohort and healthy cohort exhibited suboptimal dietary patterns, with HEI scores indicating a need for improvement, low adherence to the Mediterranean diet (aMED), and neutral-to-pro-inflammatory DII scores, with no significant between-group differences (all *p* > 0.05). Although total energy intake differed between groups (*p* = 0.04), the proportion of energy derived from UPFs (NOVA group 4) accounted for half of the total intake in both cohorts (51.3–51.8%; *p* = 0.55). Higher UPF intake was associated with lower HEI and aMED scores and higher DII scores. **Conclusions:** In this study, there were no significant differences in the dietary patterns in those with CD compared to healthy controls. The high contribution of UPFs observed in both cohorts underscores widespread suboptimal dietary quality and highlights the utility of NOVA-based food processing measures as complementary to nutrient-based assessments for understanding diet-related inflammatory burden in CD.

## 1. Introduction

The incidence of inflammatory bowel disease (IBD), a chronic inflammatory disorder of the gastrointestinal tract encompassing Crohn’s disease (CD) and ulcerative colitis (UC), has increased globally over recent decades [1,2]. In Canada, the prevalence is currently estimated at approximately 843 cases per 100,000 individuals, and is projected to increase to 1098 per 100,000 by 2035, amongst the highest in the world [1,2]. Although the etiology underlying the growing burden of IBD is multifactorial and incompletely understood, the global shift toward Westernized dietary patterns has emerged as a key modifiable environmental determinant of both disease onset and progression [3]. The Western diet is characterized by high consumption of red and processed meats, saturated fats, refined carbohydrates, and ultra-processed foods (UPFs), and is low in fibre, fruits, and vegetables [3,4,5,6,7,8]. UPFs include non-nutritive food additives such as emulsifiers and colours, thermal processing, packaging derived from plastics, and other components to enhance palatability and shelf life, contributing to nearly half of the total daily energy intake in Canada [9,10,11].

The increased consumption of UPFs has been associated with a higher incidence of IBD, as well as a lower overall diet quality, typically quantified by established dietary indices such as the Healthy Eating Index (HEI), Dietary Inflammatory Index (DII), and Alternate Mediterranean Diet score (aMED) [12,13,14,15]. While these dietary indices are widely used to assess overall diet quality and have been applied in dietary studies in IBD, they are limited to nutrient- and food-group-based evaluations and do not capture the degree of food processing within the diet. To address this limitation, Monteiro et al. developed the NOVA classification system to classify foods according to the extent and purpose of processing, rather than solely on nutrient composition [16,17]. The NOVA classification system categorizes foods into four groups: (1) unprocessed or minimally processed foods, (2) processed culinary ingredients, (3) processed foods, and (4) UPFs [18].

Since its development, NOVA has been utilized in several studies investigating UPFs and their impacts on the risk of various health outcomes, including cardiovascular disease and cancer [19,20]. Studies using the NOVA system show that UPFs, group 4, constitute a large part of the American and Canadian diet, with high UPF intake, measured as a percentage of total energy, linked to a greater risk of developing CD [9,11,21,22].

Methods for applying the NOVA classification system have been outlined by Martínez-Steele et al., who recommend a “reference approach”: food items are classified at the food code level for clearly sourced items, while mixed dishes (e.g., lasagna, curries, stir-fries, etc.) are classified at the ingredient level [18,23]. The NOVA classification of UPFs is frequently integrated with established diet quality indices, including HEI, DII, and aMED in population-based studies, demonstrating that greater UPF consumption is linked to lower overall diet quality [24,25]. Despite growing evidence linking UPF consumption to intestinal inflammation, barrier dysfunction, and microbiome perturbations, there remains limited literature characterizing UPF intake in individuals with IBD [26]. In particular, it is unclear how UPF consumption relates to established diet quality indices in IBD and whether these relationships differ from those observed in healthy individuals. Therefore, the objectives of this study are to quantify UPF consumption using the NOVA classification system, compare UPF intake between individuals with CD and a healthy cohort, and evaluate associations between UPF intake and established diet quality indices.

## 2. Materials and Methods

### 2.1. Participants

Participant data was derived from two existing cohorts of recently completed randomized controlled trials at our centre. The first cohort was recruited from the Crohn’s Disease Therapeutic Dietary Intervention (CD-TDI) and included adult patients with mild to moderately active luminal CD, defined by a Harvey–Bradshaw Index (HBI) < 16 and fecal calprotectin (FCP) > 200 μg/g. Eligible participants had no strictures or fistulae and were on stable medication therapy, including advanced biological therapies, 5-aminosalicylic acid [5-ASA], or immunosuppressants. The second cohort was involved in the Predicting Microbiome-Associated Personalized Responses to the Mediterranean Diet (MAPMed) study and consisted of healthy adults aged 20–50 years with no history of mental or physical health conditions, a normal body mass index [BMI] (20–25 kg/m^2^), and low adherence to the Mediterranean diet, defined by a Mediterranean Diet Serving Score [MDSS] of <10 points [27]. Fewer than 35% of the screened participants were deemed ineligible due to an MDSS score of ≥10 points.

### 2.2. Dietary Collection

Baseline dietary intake data was collected using the validated Automated Self-Administered 24 h Dietary Assessment Tool (ASA24^®^) [28]. Participants completed two non-consecutive dietary recalls at two different time points, during which they adhered to their habitual diet (spaced 10–12 weeks apart), with the aim of collecting four recalls per participant. Recalls were conducted by telephone by a trained research coordinator, who guided participants through the recall procedure and directly entered reported food and beverage intakes into the ASA24 system in real time to improve data collection accuracy.

Detailed information on all reported foods and beverages, including food group assignments and macronutrient and micronutrient intakes, was extracted from the ASA-24 researcher portal. Data cleaning and quality control were performed in accordance with the US National Cancer Institute [29,30]. To account for intra-individual variability in dietary intake, the median values from across the 2–4 recalls per participant were used in analyses. Macronutrient and micronutrient intakes were energy-adjusted and expressed per 1000 kcal of total energy intake.

Dietary quality indices were calculated from the 24 h dietary recalls, including the Healthy Eating Index-2015 (HEI-2015), the Alternate Mediterranean Diet score (aMED), and the Dietary Inflammatory Index (DII) [31,32,33,34].

### 2.3. NOVA Analysis

The NOVA food processing classification was conducted using an approach adapted from the reference methodology described by Martínez-Steele et al. (2023), originally developed for the analysis of dietary intake data from the National Health and Nutrition Examination Survey (NHANES), including the What We Eat in America (WWEIA) 24 h dietary recall component [18]. This framework was applied to ASA24 dietary recall data to estimate total energy intake and the proportion of energy derived from each NOVA processing category (NOVA 1–4).

ASA24 captures food-level intake data in which reported items are assigned standardized food descriptions and corresponding food codes that link to national food composition databases (e.g., the Food and Nutrient Database for Dietary Studies [FNDDS] or the Canadian Nutrient File [CNF]) for nutrient and energy estimation [35,36]. Consistent with the Martínez-Steele methodology, foods that were ready-to-eat or clearly identifiable were classified at the food-code level, whereas homemade or non-specific mixed dishes were classified at the ingredient level. Mixed and homemade dishes were systematically disaggregated into standardized recipe ingredients, each linked to a standard reference ingredient code. Ingredient-level gram weights and energy values (per 100 g) were obtained from standard reference codes, and proportional ingredient weights and associated energy contributions were calculated based on the total weight consumed at the mixed-dish food-code level. NOVA classification for mixed and homemade dishes was then assigned at the ingredient level.

NOVA group classification was applied using a semi-conservative approach informed by established NOVA classification guidelines, including those proposed by Martínez-Steele et al. (2023) and Khandpur et al. (2021) [18,23,37]. Food items with clearly identifiable sources, at either the food-code or SR ingredient-code level, were classified directly according to these published criteria. For items with ambiguous sourcing (e.g., creamers, cheeses, cured meat products), classification was guided by NOVA category definitions and ingredient lists from commonly available Canadian commercial brands.

To enhance classification reliability, NOVA classification was conducted independently by two investigators (AL and TU). The blinded classifications were compared, and discrepancies were resolved through consensus discussion. An internal consistency check for each food item and its associated NOVA classification was performed using R software (R version 4.5.1 (13 June 2025)) [38].

### 2.4. Statistical Analysis

All statistical analyses were performed using GraphPad Prism (version 10.5.0) [39]. Statistical significance was defined at a two-sided α level of 0.05. To account for multiple testing across analyses, the Bonferroni correction was applied where appropriate. Data distributions were assessed using the Shapiro–Wilk test and were found to be non-normally distributed; therefore, non-parametric tests were applied to group comparisons.

Between-group comparisons (CD vs. healthy) were conducted using the Mann–Whitney U test for continuous variables and Fisher’s exact test for categorical variables. Within-group comparisons were performed using the Kruskal–Wallis test with Dunn’s post hoc correction for multiple comparisons.

Multivariable linear regression models were used to evaluate differences in NOVA intake between groups and associations between the percentage of total energy intake derived from NOVA 4 foods and dietary quality scores, including the HEI-2015, aMED, and DII [40]. Age, BMI and total energy intake were evaluated as potential confounders and effect modifiers in group comparison analysis, and disease status was additionally assessed in the dietary quality models.

Model assumptions of normality of residuals were evaluated using residual diagnostics, including Q-Q plots and residual-versus-fitted plots. These assessments indicated that residuals were approximately normally distributed and that the assumptions of linearity and homoscedasticity were adequately met for all models. Potential confounding was evaluated by assessing whether the inclusion of covariates resulted in a ≥10% change in regression coefficients. Effect modification was assessed using the Wald test with an a priori α level of 0.05.

## 3. Results

### 3.1. Participant Demographics

A total of 112 recalls were obtained from 33 participants in the healthy cohort (mean = three recalls per participant) and 168 recalls from 64 participants in the CD cohort (mean = three recalls per participant). Participant demographics are summarized in Table 1. Briefly, participants with CD had a median age of 48.9 years, included 34 females and 30 males, and had a median body mass index (BMI) of 28.6 kg/m^2^. Baseline disease characteristics of the CD participants are presented in Table 2. Participants with CD had a median disease duration of 8.7 years (104 months) and exhibited low clinical disease activity at baseline, as reflected by a median Harvey–Bradshaw Index of 2.5. Despite relatively low systemic inflammation (median CRP 1.9 mg/L), fecal calprotectin levels remained elevated at >200 μg/g, indicating ongoing intestinal inflammation.

In comparison to CD, healthy participants were significantly younger (median age = 27.0 years; *p* < 0.001), included 18 females and 15 males, and had a significantly lower median BMI (24.1 kg/m^2^; *p* < 0.001) (Table 1).

### 3.2. Suboptimal Dietary Quality in Crohn’s Disease and Healthy Cohorts

Energy-normalized macronutrient intakes did not differ significantly between participants with CD and healthy controls (Table 3). Consumption of macronutrients (protein, carbohydrate, and fat), sodium, and saturated fat exceeded the dietary reference intake (DRI) for Canadians [47,48]. In contrast, fibre and vitamin B12 intakes were below DRI recommendations for both males and females, while folate and iron intakes fell below DRI recommendations among females.

HEI scores indicated a need for improvement in both groups (CD: median 58.9 [50.5, 71.4]; healthy controls: median 55.8 [48.7, 71.8], *p* = 0.41). Similarly, aMED scores reflected low adherence to the Mediterranean diet in both groups (CD: median 3.0 [2.5, 4.5]; healthy controls: median 3.0 [2.5, 5.0], *p* = 0.14). DII scores were consistent with neutral to pro-inflammatory dietary patterns and did not differ between groups (CD: median of 0.6 [−0.6, 1.6]; healthy controls: median of 0.1 [−1.5, 1.1], *p* = 0.12).

### 3.3. High Contribution of Ultra-Processed Foods to Energy Intake in Crohn’s Disease and Healthy Cohorts

**Figure 1:**
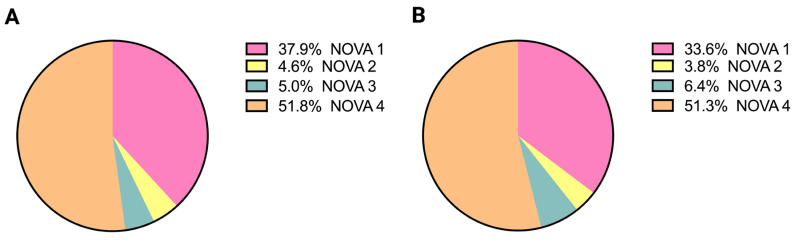
Median percentage of total energy intake across NOVA food processing categories in the Crohn’s disease and healthy cohort. (**A**) Median percentage of total energy intake (%) derived from NOVA group 1, 2, 3, and 4 foods in participants with Crohn’s disease. (**B**) Median percentage of total energy intake (%) derived from NOVA group 1, 2, 3, and 4 foods in healthy participants.

Total energy intake and the absolute and relative contributions of each NOVA food processing category are summarized in Table 4; the median percent energy distribution is shown in Figure 1. Within the CD cohort, UPF (NOVA 4) contributed the largest share of total energy intake (median: 865.3 kcal [499.9, 1391.0]) and percentage of total energy (51.8% [31.9, 57.7]), followed closely by unprocessed or minimally processed foods (NOVA 1; 785.2 kcal [557.0, 965.4]; 37.9% [27.9, 50.1]). Within-group comparisons (Bonferroni-adjusted α = 0.008) demonstrated that NOVA 4 foods contributed significantly more total energy and a greater proportion of energy than NOVA 2 and NOVA 3 foods (all *p* < 0.001), while no significant differences were observed between NOVA 1 and NOVA 4 contributions within the CD cohort.

Among healthy controls, NOVA 4 foods similarly accounted for the greatest total energy intake (1146.0 kcal [861.2, 1441.0]) and percentage of total energy (51.3% [42.4, 56.2]), contributing significantly more energy and a higher proportion of total energy than NOVA 2 and 3 categories (all *p* ≤ 0.001, Bonferroni-adjusted α = 0.008). However, no significant differences were observed between NOVA 1 and NOVA 4 contributions within the healthy cohort.

After Bonferroni correction, no significant between-group differences were detected for total energy, or the proportion of energy derived from each NOVA category. However, total energy intake differed significantly between groups in a single comparison (1988.0 vs. 2296.0 kcal; *p* = 0.04).

Crude comparisons of percent energy intake from NOVA food groups revealed no significant differences between CD and healthy participants (Table 5). Effect modification by age, BMI or total energy intake was not observed; however, both age and BMI were identified as confounders. After adjustment for age and BMI using multivariable linear regression, CD patients consumed a higher percentage of energy from NOVA 2 food than healthy controls (adjusted difference: 2.6%, 95% CI 0.5, 4.7; *p* = 0.02), although this difference did not reach the Bonferroni-corrected significance threshold of α = 0.0125. There were no significant differences in percent energy intake of NOVA 1, 3 and 4 foods between groups after adjustment.

### 3.4. Relationships Between NOVA Group 4 Intake and Dietary Quality and Inflammatory Indices

Associations between the percentage of total energy intake derived from UPFs (NOVA 4) and dietary quality indices are shown in Table 6. Effect modification by age, BMI, total energy intake and disease status was not observed; however, age, BMI, and disease status were included as covariates in the models.

After adjustment, a higher proportion of energy from NOVA 4 foods was consistently associated with lower overall diet quality, as reflected by both HEI and aMED scores. Specifically, each 1% increase in NOVA 4 intake was associated with a 0.38 point decrease in HEI scores (95% CI −0.51 to −0.25, *p* < 0.001) and a 0.035 point decrease in aMED scores (95% CI −0.05 to −0.02, *p* < 0.001). Higher NOVA 4 intake was associated with a more pro-inflammatory dietary profile, with each 1% increase in NOVA 4 intake corresponding to a 0.027point increase in DII (95% CI 0.004 to 0.05, *p* = 0.02). Nonetheless, the association between NOVA 4 and DII did not meet the Bonferroni-corrected significance threshold of α = 0.0167.

## 4. Discussion

This study provides a characterization of dietary intake, food processing patterns and dietary quality in a cohort of individuals with CD compared with a healthy adult population, integrating the NOVA food processing classification system with established dietary quality indices (HEI-2015, aMED and DII). Collectively, our findings demonstrate that both cohorts consume diets of suboptimal quality, with half of total energy intake derived from UPFs, and no observed difference between healthy individuals and individuals with CD. These findings underscore the widespread adoption of Westernized, UPF-dominated dietary patterns across our population in Canada, irrespective of disease status.

An emerging body of evidence from systematic reviews and meta-analyses has demonstrated that the risk of CD is positively associated with low-quality dietary patterns characterized by high intakes of UPFs, refined carbohydrates, red and processed meats, and added sugars, alongside low intake of dietary fibre, fruit, and vegetables [3,49]. This trend is reinforced by the observed macronutrient and nutrient profiles of both cohorts, in which protein, carbohydrate, total fat, saturated fat, and sodium intakes exceeded the DRI, whereas fibre intake remained well below recommended levels. Although energy-normalized nutrient profiles identify whether intake targets are met or exceeded, they do not capture qualitative aspects of diet, such as carbohydrate or protein source. Incorporating dietary quality indices alongside the NOVA food-processing classification system enables a more nuanced and comprehensive evaluation of overall dietary quality.

Greater adherence to healthy dietary indices, captured by the HEI, has been inversely associated with CD risk and may correlate with disease severity and symptom burden [50,51,52,53,54]. The “needs improvement” HEI scores observed in the CD cohort in the present study are consistent with this literature and reinforce previously described dietary trends in CD. Notably, HEI scores did not differ significantly between CD and healthy participants, which suggests that suboptimal diet quality may reflect broader population-level behaviours rather than disease-specific patterns. The HEI scores of both healthy and CD participants align with prior analyses of the 2015 Canadian Community Health Survey, which reported a mean HEI score of 59 [55]. Given the established associations between poor diet quality, IBD incidence, and adverse disease outcomes, the similarities between national averages highlights that suboptimal diet quality is widespread rather than disease specific. There is a need to integrate dietary improvements at both the population health and clinical care levels for the prevention and treatment of diseases, such as IBD.

A similar lack of differentiation between cohorts was observed with respect to the dietary inflammatory potential. DII scores were comparable between healthy participants and those with CD, indicating similar inflammatory dietary profiles across groups. While evidence linking DII scores to IBD risk is mixed, higher DII scores (more pro-inflammatory) have been associated with increased disease activity and symptom burden in CD [52,56,57]. The present findings are consistent with prior observational studies reporting no significant difference in dietary inflammatory load between healthy individuals and those living with CD [51]. Collectively, these data suggest that pro-inflammatory dietary patterns may be widespread in the general population; however, the clinical relevance may be amplified in individuals with CD due to heightened susceptibility to diet–immune–microbiome interactions.

Adherence to the Mediterranean diet (aMED) did not differ between groups. Both healthy and those with CD demonstrated low adherence to the Mediterranean-style diet pattern, further reinforcing the observed suboptimal dietary profiles across groups. This finding is consistent with previous observational studies reporting poor adherence to Mediterranean-style dietary patterns among individuals with CD [58,59]. This is clinically relevant, given evidence that high adherence to a Mediterranean diet pattern is associated with reduced risk of CD, and improved clinical and biochemical outcomes, including reduced HBI and fecal calprotectin [50,53,60]. These findings highlight a potentially actionable gap in dietary quality among both individuals with CD and healthy adults.

Despite growing evidence linking UPF consumption to increased IBD risk and worse disease outcomes, UPF intake did not differ significantly between cohorts, with NOVA group 4 foods contributing half of total energy intake in both groups [13,14,22,26,61,62]. This pattern parallels national Canadian dietary intake data and raises concern that individuals with CD may not substantially modify dietary behaviours following diagnosis [9,11,63]. Importantly, higher UPF intake was consistently associated with poorer dietary quality, including lower HEI and Mediterranean diet adherence and higher dietary inflammatory potential, reinforcing the close relationship between food processing and overall diet quality.

Potential confounding factors were evaluated. Although medication use was stable, steroid use was controlled for and did not significantly influence the results. Age and BMI differed between cohorts, likely reflecting the MAPMed recruitment criteria restricting healthy participants to a BMI of 20–25 kg/m^2^ and an age of 20–50 years. BMI and age were identified as confounders in the between-group analyses of percent energy intake from each NOVA category, as well as for NOVA group 4 intake, HEI score, aMED score, and DII score. Total energy intake was evaluated and found not to confound the results.

Methodological considerations are important when interpreting these findings. Studies using 24 h dietary recalls combined with NOVA-based classification have demonstrated more consistent associations between UPF intake and IBD risk than those relying on FFQs alone, supporting the sensitivity of the present approach [61]. The NOVA classification is subject to inherent ambiguity, particularly for mixed or home-prepared dishes, contributing to recognized criticism regarding inter-study variability and reproducibility. Nevertheless, NOVA-based classification approaches have shown more consistent associations between UPF intake and disease risk than proxy food-group or dietary-pattern methods [61]. Although this limitation applies to the present study, ingredient-level classification for mixed dishes, use of established definitions, and evaluation of current Canadian market products likely minimized its impact on relative intake estimates and primary conclusions [23]. Consistency with national dietary survey data, indicating that UPFs contribute approximately 45–48% of total energy intake in Canada, provides evidence that although study inter-variability of classification exists, on average, UPF consumption is contributing nearly half of the intake of the Canadian population, both healthy and diseased populations alike [9,11,63].

The increasing adoption of NOVA classification protocols, such as those proposed by Martínez-Steele et al. [18], represents an important methodological advance. These approaches enable food-level and ingredient-level evaluation of dietary intake across all NOVA 1–4 categories, rather than relying on proxy food groups or limited UPF definitions (e.g., fast food alone) [61]. In this study, we adapted the reference methodology to Canadian ASA24 data, providing a robust and reproducible characterization of UPF intake in both a CD and a healthy cohort.

Nevertheless, several limitations warrant consideration. The NOVA classification system is inherently subjective, particularly for mixed or home-prepared foods, and standardized NOVA protocols for Canadian dietary recall datasets remain limited [9,18]. Additionally, ASA24 dietary recalls lack detailed brand- and source-specific information, necessitating assumptions about commonly available products, which may introduce classification uncertainty. Given the retrospective nature of this analysis, it was not possible to resolve the classification subjectivity of mixed or home-prepared dishes or to obtain brand-specific information. As with all self-reported dietary assessment tools, ASA24 is also subject to recall bias and measurement error. Finally, although minimal differences were observed between cohorts, the small sample size of the healthy cohort should be noted. Larger studies are warranted to further evaluate differences in dietary patterns between CD and healthy populations.

## 5. Conclusions

In conclusion, this study demonstrates that both individuals with CD and healthy adults consume diets characterized by high UPF intake and suboptimal dietary quality, with limited differentiation between cohorts. The consistently high contribution of NOVA group 4 foods, alongside low adherence to healthy dietary patterns such as the Mediterranean diet and comparable dietary inflammatory profiles, underscores the pervasive influence of Westernized dietary habits across populations, irrespective of disease status. The observation that both patients with Crohn’s disease and healthy adults demonstrate suboptimal dietary quality and high intake of UPFs is concerning, given its association with an increased risk of IBD and inadequate disease control. Importantly, the observed associations between greater ultra-processed food intake and poorer dietary quality across both cohorts highlight food processing as a key determinant of diet quality. Together, these findings identify UPF reduction and promotion of minimally processed, nutrient-dense dietary patterns as important and potentially modifiable targets for improving diet quality and supporting disease management in CD, warranting further evaluation in larger longitudinal and intervention-based studies. Further, these findings are particularly relevant in the Canadian context, where the prevalence of IBD is high. Further investigation is warranted to elucidate the relationships between consumption of ultra-processed foods and dietary quality indices across diverse geographic and cultural settings, including regions with historically low but rapidly increasing IBD incidence.

## Figures and Tables

**Table 1:** Baseline demographics and diet quality indices for individuals with Crohn’s disease and healthy controls.

	Crohn’s Disease (*n* = 64) Median [IQR] or *n* (%)	Healthy Controls (*n* = 33) Median [IQR] or *n* (%)	*p*-Value ^#^
Sex (Female, %)	34 (53.1)	18 (54.6)	>0.99
Age (Years)	48.9 [34.9, 55.9]	27.0 [23.0, 30.5]	<0.001
Body Mass Index (kg/m^2^)	28.6 [24.1, 31.2]	24.2 [22.2, 24.9]	<0.001
Healthy Eating Index-2020 ^1^	58.9 [50.5, 71.4]	55.8 [48.7, 71.8]	0.41
Alternate Mediterranean Diet Score ^2^	3.0 [2.5, 4.5]	3.0 [2.5, 5.0]	0.14
Dietary Inflammatory Index ^3^	0.6 [–0.6, 1.6]	0.1 [–1.5, 1.1]	0.12

Abbreviations: Interquartile range (IQR). ^1^ The Healthy Eating Index evaluates how closely individuals adhere to American or Canadian dietary guidelines, with higher scores reflecting better diet quality [41]. Scores range from 0 to 100, with scores equal or below 50 considered poor diet quality, scores between 51 and 80 indicating a need for improvement, and scores above 80 considered good diet quality [42,43]. ^2^ The Alternate Mediterranean Diet Score evaluates how closely individuals adhere to the Mediterranean diet pattern. Scores range from 0 to 9, with a higher score indicating greater adherence. Scores from 0 to 3 indicate low adherence, 4 to 5 moderate adherence, and 6 to 9 high adherence [44,45,46]. ^3^ The Dietary Inflammatory Index classifies individuals’ diets according to their inflammatory potential. The score ranges from strongly pro-inflammatory at 7.89 to strongly anti-inflammatory at −8.87 [33]. ^#^ Between-group comparisons by sex were performed using Fisher’s exact test. Between-group differences in age, BMI, HEI, aMED, and DII scores were assessed using the Mann–Whitney U test. (*p* < 0.05).

**Table 2:** Baseline clinical disease characteristics of Crohn’s Disease participants.

	Crohn’s Disease (*n* = 64) Median [IQR] or *n* (%)
Disease duration (months)	104.3 [16.5, 249.9]
Medication use	
Steroids (%)	5.0 (7.8%)
Biologics (%)	20.0 (31.3%)
5-Aminosalicylic Acid (%)	16.0 (25.0%)
Immunosuppressants (%)	13.0 (20.3%)
Harvey–Bradshaw Index	2.5 [1.0, 4.8]
Fecal calprotectin (μg/g)	246.0 [119.0, 558.8]
C-Reactive protein (mg/L)	1.9 [0.8, 4.5]

Abbreviations: Interquartile range (IQR). Values are presented as median [interquartile range] or number (%).

**Table 3:** Energy-normalized macronutrient and micronutrient intake among participants with Crohn’s disease and healthy controls.

Macronutrient and Nutrients	Dietary Reference Intake ^‡^	Crohn’s Disease ^a^ Median [IQR]	Healthy Controls ^b^ Median [IQR]	*p*-Value ^#^
Protein (g/1000 kcal)	M: 22.4 F: 25.6	44.5 [35.8, 52.5]	43.2 [39.7, 49.9]	0.92
Carbohydrate (g/1000 kcal)	M: 52.0 F: 72.2	112.2 [99.4, 131.5]	107.1 [99.0, 118.0]	0.15
Fat (g/1000 kcal)	M: 30.0 F: 41.7	40.5 [35.4, 47.3]	42.2 [38.9, 47.1]	0.34
Saturated Fatty Acids (g/1000 kcal)	M: 8.0 F: 11.1	12.2 [10.6, 14.4]	13.0 [11.3, 16.2]	0.24
Fibre (g/1000 kcal)	M: 13.9 F: 15.2	8.8 [7.4, 11.4]	8.4 [6.1, 12.0]	0.52
Sugar (g/1000 kcal)	M: 40.0 F: 55.6	41.4 [33.3, 51.7]	40.6 [31.1, 51.4]	0.63
Sodium (mg/1000 kcal)	M: 600.0 F: 833.0	1631.0 [1359.0, 1878.0]	1658.0 [1424.0, 1837.0]	0.57
Vitamin B12 (μg/1000 kcal)	M: 1.0 F: 1.3	2.1 [1.3, 2.7]	2.0 [1.5, 2.5]	0.91
Zinc (mg/1000 kcal)	M: 4.4 F: 4.4	5.3 [4.6, 6.4]	5.3 [5.0, 6.0]	0.96
Iron (mg/1000 kcal)	M: 3.2 F: 10.0	6.3 [5.5, 7.6]	6.7 [6.1, 7.2]	0.27
Folate (μg/1000 kcal)	M: 160.0 F: 222.0	178.1 [150.1, 233.6]	179.8 [146.3, 223.6]	0.96

Abbreviations: M: male; F: female; Interquartile range (IQR). ^‡^ Dietary Reference Intake provided per 1000 kcal [47,48]. ^a^ Participants *n* = 64. ASA-24 Dietary Recalls *n* = 168. ^b^ Participants *n* = 33. ASA-24 Dietary Recalls *n* = 112. ^#^ Between-group comparisons (Crohn’s disease vs. Healthy) of macronutrients and nutrients were performed using the Mann–Whitney U test (*p* < 0.05).

**Table 4:** Total energy intake and percentage contribution of NOVA food processing categories among participants with CD and healthy controls.

	Crohn’s Disease ^a^ Median [IQR]	Healthy Controls ^b^ Median [IQR]	*p*-Value ^#^
Total energy intake (kcal)	1988.0 [1474.0, 2525.0]	2296.0 [1991.0, 2693.0]	0.04
NOVA 1 energy (kcal)	785.2 [557.0, 965.4]	683.6 [553.7, 1038.0]	0.91
NOVA 1 (% energy)	37.9 [27.9, 50.1]	33.6 [23.9, 42.2]	0.07
NOVA 2 energy (kcal)	90.7 [41.4, 156.0]	86.9 [30.7, 134.5]	0.45
NOVA 2 (% energy)	4.6 [2.0, 8.1]	3.8 [1.0, 6.0]	0.17
NOVA 3 energy (kcal)	92.4 [23.47, 237.1]	149.2 [71.5, 384.8]	0.06
NOVA 3 (% energy)	5.0 [1.2, 11.6]	6.4 [3.2, 16.2]	0.11
NOVA 4 energy (kcal)	865.3 [499.9, 1391.0]	1146.0 [861.2, 1441.0]	0.05
NOVA 4 (% energy)	51.8 [31.9, 57.7]	51.3 [42.4, 56.2]	0.55

Abbreviations: Interquartile range (IQR); food processing classification system (NOVA). ^#^ Between-group comparisons (Crohn’s disease vs. healthy controls) of total energy intake and percentage contribution of each NOVA food group were performed using the Mann–Whitney U test. Bonferroni correction was applied to comparisons across NOVA food processing categories α = 0.0125 (0.05/4). Total energy intake was evaluated as a single comparison (α = 0.05). Footnotes: ^a^ Crohn’s disease participants: *n* = 64. ASA-24 Dietary Recalls *n* = 168. ^b^ Healthy controls: *n* = 33. ASA-24 Dietary Recalls *n* = 112.

**Table 5:** Unadjusted and adjusted difference in percentage of energy intake from NOVA food groups between participants with CD and healthy controls.

Analysis ^†^	Unadjusted Difference in Percent (%) Energy Intake (95% CI)	Adjusted Difference in Percent (%) Energy Intake (95% CI)	*p*-Value ^#^
NOVA 1%	6.1 (−0.2 to 12.3)	7.6 (−0.5 to 15.7)	0.07
NOVA 2%	1.2 (−0.4 to 2.9)	2.6 (0.5 to 4.7)	0.02
NOVA 3%	−2.4 (−6.2 to 1.3)	−2.7 (−7.6 to 2.2)	0.28
NOVA 4%	−4.3 (−10.8 to 2.1)	−5.5 (−13.9 to 3.0)	0.20

^†^ Multivariable linear regression was used to assess the difference in percentage of energy intake from NOVA food groups between participants with Crohn’s disease and the healthy controls, adjusting for age and BMI. Healthy controls were used as the reference group. Regression coefficients represent the difference in percent energy intake between groups. ^#^ Bonferroni correction was applied to account for multiple comparisons across the four NOVA food groups. The adjusted significance threshold was α = 0.0125 (0.05/4). *p*-values below this threshold are considered statistically significant.

**Table 6:** Multivariable linear regression analyses examining associations between the percentage of total energy intake from ultra-processed foods (NOVA group 4) and dietary quality indices in healthy controls and participants with Crohn’s disease.

Analysis ^†^	β	CI	*p*-Value ^#^
HEI~NOVA 4 (% intake)	−0.38	−0.51 to −0.25	<0.001
aMED~NOVA 4 (% intake)	−0.035	−0.05 to −0.02	<0.001
DII~NOVA 4 (% intake)	0.027	0.004 to 0.05	0.02

Abbreviations: HEI, Healthy Eating Index; aMED, Alternate Mediterranean Diet score; DII, Dietary Inflammatory Index; CI, confidence interval. ^†^ Multivariable linear regression was used to assess associations between the percentage of total energy intake derived from ultra-processed foods (NOVA group 4) and dietary quality indices, adjusting for age, BMI and disease status. NOVA 4 intake was modelled as a continuous variable, with regression coefficients (β) representing the change in dietary index score per 1% increase in energy intake from NOVA 4 foods. ^#^ Statistical significance was evaluated using two-sided *p*-values. Bonferroni correction was applied for the three dietary quality outcomes, resulting in an adjusted significance threshold of α = 0.0167 (0.05/3). *p*-values below this threshold are considered statistically significant.

## Data Availability

The data presented in this study are available upon request from the corresponding author due to privacy and ethical restrictions.
